# Susceptibility of Colistin-Resistant, Gram-Negative Bacteria to Antimicrobial Peptides and Ceragenins

**DOI:** 10.1128/AAC.00292-17

**Published:** 2017-07-25

**Authors:** Marjan M. Hashemi, John Rovig, Scott Weber, Brian Hilton, Mehdi M. Forouzan, Paul B. Savage

**Affiliations:** a Department of Chemistry and Biochemistry, Brigham Young University, Provo, Utah, USA; b Department of Chemical Engineering, Brigham Young University, Provo, Utah, USA

**Keywords:** colistin, resistant, antimicrobial peptides, ceragenin, Gram-negative bacteria

## Abstract

The susceptibility of colistin-resistant clinical isolates of Klebsiella pneumoniae to ceragenins and antimicrobial peptides (AMPs) suggests that there is little to no cross-resistance between colistin and ceragenins/AMPs and that lipid A modifications are found in bacteria with modest changes in susceptibility to ceragenins and with high levels of resistance to colistin. These results suggest that there are differences in the resistance mechanisms to colistin and ceragenins/AMPs.

## TEXT

The continuous emergence of drug-resistant bacteria has led to dire predictions of a possible “postantibiotic” era in which common infections will not be treatable with the current arsenal of antibiotics (1, 2). Of particular concern are Gram-negative bacteria, because these organisms are inherently resistant to many antibiotics due to the permeability barrier provided by their outer membranes and the efflux pumps located therein (3, 4). To treat infections from Gram-negative bacteria, clinicians are increasingly using colistin, a member of the polymyxin family of antibiotics (5). Colistin is considered the antibiotic of last resort because, while it has side effects, including nephrotoxicity and ototoxicity, it is broadly active against Gram-negative bacteria (6, 7). Isolation of colistin-resistant bacteria in many countries underscores the need for development of novel strategies for targeting Gram-negative bacteria, including drug-resistant strains (8).

**FIG 1:**
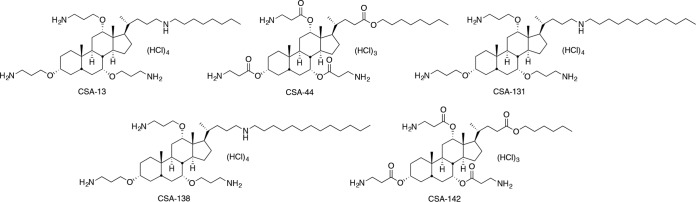
Structure of ceragenins CSA-13, CSA-44, CSA-131, CSA-138, and CSA-142.

Endogenous antimicrobial peptides (AMPs) have played an important role in innate immunity for eons (9), and there have been efforts to use AMPs clinically. Challenges for clinical use of peptide therapeutics include the relatively high costs of large-scale production and the susceptibility of AMPs to degradation by proteases (10). We have developed a class of small molecules, termed ceragenins (Fig. 1), that circumvent these challenges while maintaining the same general mechanism of bactericidal activity of AMPs. Ceragenins can be prepared on a large scale, and because they are not peptide based, they are not substrates for proteases. As mimics of AMPs, ceragenins display broad-spectrum antibacterial activity, including potent antibiofilm activity (11–13). In *in vivo* studies involving medical devices and bone regrowth, ceragenins are effective in eliminating bacterial challenges, and local administration is well tolerated (14–16).

Consideration of the common structural features of colistin, AMPs, and ceragenins (multiple cationic groups, substantial hydrophobic character, and interaction with bacterial membranes) leads to three questions. (i) Are colistin-resistant bacteria also resistant to AMPs and ceragenins? (ii) Does generation of resistance to colistin occur at the same rate as potential generation of resistance to AMPs and ceragenins? (iii) Since the primary mechanism for bacterial resistance to AMPs and ceragenins is through modification of the lipid A portion of lipopolysaccharide (LPS) (17–19), how important are these modifications in the resistance of Gram-negative bacteria to colistin, AMPs, and ceragenins?

To address these questions, we compared the susceptibility of colistin-resistant clinical isolates of Klebsiella pneumoniae to colistin, representative AMPs (LL-37, cecropin A, and magainin 1), and the representative ceragenins shown in Fig. 1. Our initial focus on K. pneumoniae was due to its known pathogenicity and its ability to transfer resistance genes to other Gram-negative bacteria (20, 21). We and our collaborators previously found ceragenins to be active against other colistin-resistant bacteria (19, 22), and we have extended these observations to clinical isolates of K. pneumoniae using additional, later-generation ceragenins. MICs and minimum bactericidal concentrations (MBCs) were determined using a broth microdilution method (CLSI protocol, with Mueller-Hinton [MH] substituted for cation-adjusted MH) (23). Colistin-resistant clinical isolates of K. pneumoniae gave MICs of 16 to 200 μg/ml with colistin, while a susceptible strain (ATCC 13883) gave an MIC of 2 μg/ml (Table 1). MICs of LL-37 and magainin 1 were relatively high against the reference strain as well as the clinical isolates; MICs were lower and less varied with cecropin A. With the ceragenins, MICs with the susceptible strain were relatively low (1 to 3 μg/ml), and only small changes in MICs were observed with colistin-resistant isolates. MBCs with CSA-44 and CSA-131 were 2 to 10 μg/ml, demonstrating bactericidal rather than bacteriostatic activity, and colistin resistance did not significantly impact the MBCs of the ceragenins.

**TABLE 1:** MICs of colistin, AMPs, and CSAs against a standard strain of K. pneumoniae (ATCC 13883) and colistin-resistant clinical isolates

K. pneumoniae strain	MICs (MBCs) (μg/ml) for:								
	Colistin	LL-37	Cecropin A	Magainin 1	CSA-13	CSA-44	CSA-131	CSA-138	CSA-142
ATCC 13883	2.0	32	2.0	64	2.0	1.0 (2.0)	1.0 (2.0)	3.0	3.0
ARLG-1127	32	64	2.0	64	2.0	1.0 (2.0)	1.0 (2.0)	2.0	2.0
ARLG-1340	100	100	NM^*a*^	NM	2.0	1.0 (2.0)	1.0 (6.0)	3.0	4.0
ARLG-1349	16	64	4.0	64	2.0	1.0 (2.0)	3.0 (4.0)	3.0	8.0
ARLG-1360	64	100	4.0	150	2.0	1.0 (2.0)	2.0 (6.0)	6.0	6.0
ARLG-1389	200	100	4.0	200	6.0	2.0 (2.0)	3.0 (10)	8.0	8.0
ARLG-1406	64	64	4.0	100	3.0	1.0 (3.0)	3.0 (8.0)	6.0	16

a NM, not measured.

**FIG 2:**
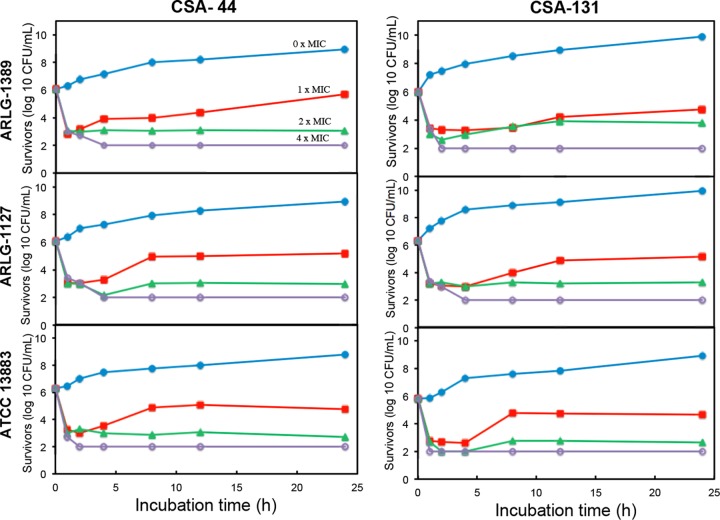
Rates of bactericidal activity of ceragenins are similar among colistin-resistant and colistin-susceptible strains. Time-kill curves with CSA-44 and CSA-131 against colistin-resistant K. pneumoniae strains (ARLG-1127 and ARLG-1389) and colistin-susceptible strain ATCC 13883. Detection limit is 2 logs (CFU/ml).

To quantify rates of bactericidal activity, time-kill assays were performed with CSA-44 and CSA-131 against the colistin-resistant strains ARLG-1127 and ARLG-1389 and compared to a susceptible strain of K. pneumoniae (Fig. 2). For these assays, the protocol for MIC measurement was used, and aliquots (10 μl) were removed at varied intervals, plated on nutrient agar, and incubated (24). At 2× MIC for both ceragenins, the inoculum was decreased by at least 3 logs within 2 h. At 4× MIC, the inoculum was decreased to the detection limit (2 logs) within the same time frame. These assays revealed that there are only minor differences in the kinetics of bactericidal activity among the colistin-resistant and colistin-susceptible strains, again suggesting that colistin resistance does not significantly influence susceptibility to ceragenins.

We compared the relative rates at which K. pneumoniae (ATCC 13883) and other Gram-negative bacteria (Pseudomonas aeruginosa [ATCC 27853] and Acinetobacter baumannii [ATCC 19606]) become resistant to colistin and ceragenin CSA-131 by serially exposing these organisms to both antimicrobials and monitoring susceptibility. MICs (at 24 h) for the strains were determined, and bacterial populations growing at the highest concentrations of the antimicrobials were used to inoculate fresh medium. This process was repeated every 18 to 24 h. Concentrations of the antimicrobials were adjusted to allow determination of MICs (19). This process was repeated for 10 periods (of 24 h each) with colistin, with MICs rising from 1 to 2 μg/ml to ≥350 μg/ml, and for 30 days with CSA-131. Resulting MICs are shown in Table 2, along with the susceptibility of the resulting bacteria to colistin, CSA-131, and representative AMPs. Serial exposure to colistin, resulting in the generation of highly resistant organisms, caused little or no change in MICs with CSA-131. Some changes in MICs were observed with the AMPs against colistin-resistant organisms (1.3- to 8-fold increases). Serial exposure to CSA-131 resulted in increases of MICs from 1 to 2 μg/ml to 2 to 8 μg/ml and resulted in increased MICs with AMPs (2.3- to 12-fold increases).

**TABLE 2:** MICs of colistin, CSA-131, LL-37, magainin 1, and cecropin A with susceptible standard strains of K. pneumoniae, A. baumannii, and P. aeruginosa and with strains serially exposed to colistin or CSA-131

Strain	MICs (μg/ml) for:				
	Colistin	CSA-131	LL-37	Magainin 1	Cecropin A
K. pneumoniae ATCC 13883	2.0	1.0	32	64	2.0
Serially exposed to colistin^*a*^	350	1.5	64	82	16
Serially exposed to CSA-131^*b*^	32	8.0	100	150	24
A. baumannii ATCC 19606	1.0	2.0	16	32	4.0
Serially exposed to colistin^*a*^	400	2.0	64	100	16
Serially exposed to CSA-131^*b*^	32	2.0	128	150	32
P. aeruginosa ATCC 27853	1.0	2.0	32	64	4.0
Serially exposed to colistin^*a*^	350	2.0	64	100	8.0
Serially exposed to CSA-131^*b*^	32	4.0	100	150	16

a Ten days of exposure.

b Thirty days of exposure.

In a previous study, we found that serial exposure of Gram-negative bacteria to ceragenin CSA-13 resulted in increased MICs and modifications to the lipid A portion of lipopolysaccharide (19). Lipid A is a primary target of colistin (25), ceragenins (11), and AMPs (26), and lipid A modifications were observed as mechanisms for the generation of resistance to these antimicrobials (26–30). To determine if colistin resistance and serial exposure to CSA-131 result in comparable lipid A modifications (phosphate ester formation with 4-aminoarabinose and/or ethanolamine), lipid A was isolated from three colistin-resistant clinical isolates of K. pneumoniae, and bacteria were serially exposed to colistin or CSA-131. Lipid A was isolated using the TRI reagent method (31) and analyzed via mass spectrometry (electrospray ionization, negative-ion mode, Agilent 6230 series time-of-flight spectrometer). Lipid A from the parent strain (K. pneumoniae [ATCC 13883]) showed the expected masses lacking 4-aminoarabinose and ethanolamine, while lipid A from each of the clinical isolates and from bacteria serially exposed to colistin or CSA-131 showed these modifications (Table 3 [mass spectra are shown in the supplemental material]), as expected from activation of two-component systems (e.g., PhoP/PhoQ and PmrA/PmrB) (26–30). Additions of fatty acids were also observed. Comparable masses were observed for parent and modified lipid A (27, 32, 33). Susceptibility of these strains to colistin or CSA-131 varies dramatically, and yet there are modifications to lipid A common to these organisms. These modifications to lipid A may impact the activities of colistin and CSA-131 differently; alternatively, there may be other mechanisms of resistance with these organisms (e.g., loss of lipopolysaccharide) (34) that provide high levels of colistin resistance without influencing susceptibility to CSA-131.

**TABLE 3:** Masses of isolated lipid A from colistin-susceptible (ATCC 13883) and colistin-resistant strains of K. pneumoniae and bacteria serially exposed to colistin and CSA-131

K. pneumoniae strain	Observed lipid A mass (*m*/*z*)	Mass of parent lipid A structures (*m*/*z*)	Additions to parent lipid A structures
ATCC 13883^*c*^	1,795		
	1,840		
	1,853		
	1,910		
ARLG-1389	2,023	1,840	Lauric acid
	2,209	1,840	Aminoarabinose and palmitic acid
ARLG-1349	2,021	1,840	Lauric acid
	2,209	1,840	Aminoarabinose and palmitic acid
ARLG-1360	2,034	1,910	Ethanolamine
ATCC 13883 serially exposed to colistin^*a*^	1,795	1,795	
	1,987	1,840	Aminoarabinose and hydroxyl group
ATCC 13883 serially exposed to CSA-131^*b*^	1,840	1,840	
	2,021	1,840	Lauric acid
	2,152	1,840	Aminoarabinose and lauric acid

a Ten days of exposure.

b Thirty days of exposure.

c ATCC 13883 is the fully susceptible strain, so no modifications to lipid A are identified (this is the source of the parent lipid A structures).

Considering the common structural features of colistin, AMPs, and ceragenins, the issue of cross-resistance arises. Among colistin-resistant isolates and strains generated by serial exposure to colistin, MICs increase up to several hundredfold compared to susceptible strains, while up to 5-fold increases in MICs were observed with the AMPs and ceragenins tested. However, in some cases, MICs of ceragenins were the same with both colistin-susceptible and -resistant bacteria. While specific mechanisms for resistance of Gram-negative bacteria to colistin have been identified, multiple mechanisms likely influence resistance (35). At least one of these mechanisms causes very high resistance to colistin but does not appear to impact, to the same extent, susceptibility to AMPs and ceragenins. These observations are consistent with the reported susceptibility of colistin-resistant bacteria to a variety of AMPs (36, 37). The fact that lead ceragenins (CSA-44 and CSA-131) retain bactericidal activity against highly colistin-resistant bacteria provides further support for development of these compounds as broad-spectrum antibacterial agents in multiple potential clinical applications.

## Supplementary Material

Supplemental material

## References

[1] Rojas LJ, Salim M, Cober E, Richter SS, Perez F, Salata RA, Kalayjian RC, Watkins RR, Marshall S, Rudin SD 2017 Colistin resistance in carbapenem-resistant *Klebsiella pneumoniae*: laboratory detection and impact on mortality. Clin Infect Dis 64:711–718. doi:10.1093/cid/ciw805. 27940944 PMC5850634

[2] McGann P, Snesrud E, Maybank R, Corey B, Ong AC, Clifford R, Hinkle M, Whitman T, Lesho E, Schaecher KE 2016 *Escherichia coli* harboring mcr-1 and blactx-m on a novel IncF plasmid: first report of mcr-1 in the United States. Antimicrob Agents Chemother 60:4420–4421. doi:10.1128/AAC.01103-16. 27230792 PMC4914657

[3] Aminlari L, Hashemi MM, Aminlari M 2014 Modified lysozymes as novel broad spectrum natural antimicrobial agents in foods. J Food Sci 79:R1077–1090. doi:10.1111/1750-3841.12460. 24837015

[4] Savage PB 2001 Multidrug-resistant bacteria: overcoming antibiotic permeability barriers of Gram-negative bacteria. Ann Med 33:167–171. doi:10.3109/07853890109002073. 11370769

[5] Li J, Rayner CR, Nation RL, Owen RJ, Spelman D, Tan KE, Liolios L 2006 Heteroresistance to colistin in multidrug-resistant *Acinetobacter baumannii*. Antimicrob Agents Chemother 50:2946–2950. doi:10.1128/AAC.00103-06. 16940086 PMC1563544

[6] Cheng YH, Lin TL, Pan YJ, Wang YP, Lin YT, Wang JT 2015 Colistin resistance mechanisms in *Klebsiella pneumoniae* strains from Taiwan. Antimicrob Agents Chemother 59:2909–2913. doi:10.1128/AAC.04763-14. 25691646 PMC4394772

[7] Lim LM, Ly N, Anderson D, Yang JC, Macander L, Jarkowski A, Forrest A, Bulitta JB, Tsuji BT 2010 Resurgence of colistin: a review of resistance, toxicity, pharmacodynamics, and dosing. Pharmacotherapy 30:1279–1291. doi:10.1592/phco.30.12.1279. 21114395 PMC4410713

[8] Castanheira M, Griffin MA, Deshpande LM, Mendes RE, Jones RN, Flamm RK 2016 Detection of mcr-1 among *Escherichia coli* clinical isolates collected worldwide as part of the SENTRY antimicrobial surveillance program during 2014-2015. Antimicrob Agents Chemother 60:5623–5624. doi:10.1128/AAC.01267-16. 27401568 PMC4997847

[9] Cirioni O, Giacometti A, Ghiselli R, Bergnach C, Orlando F, Silvestri C, Mocchegiani F, Licci A, Skerlavaj B, Rocchi M 2006 LL-37 protects rats against lethal sepsis caused by Gram-negative bacteria. Antimicrob Agents Chemother 50:1672–1679. doi:10.1128/AAC.50.5.1672-1679.2006. 16641434 PMC1472226

[10] Jenssen H, Hamill P, Hancock REW 2006 Peptide antimicrobial agents. Clin Microb Rev 19:491–511. doi:10.1128/CMR.00056-05. PMC1539102 16847082

[11] Lai X-Z, Feng Y, Pollard J, Chin JN, Rybak MJ, Bucki R, Epand RF, Epand RM, Savage PB 2008 Ceragenins: cholic acid-based mimics of antimicrobial peptides. Acc Chem Res 41:1233–1240. doi:10.1021/ar700270t. 18616297

[12] Chin JN, Jones RN, Sader HS, Savage PB, Rybak MJ 2008 Potential synergy activity of the novel ceragenin, CSA-13, against clinical isolates of *Pseudomonas aeruginosa*, including multidrug-resistant *P. aeruginosa*. J Antimicrob Chemother 61:365–370. 18079128 10.1093/jac/dkm457

[13] Niemirowicz K, Surel U, Wilczewska AZ, Mystkowska J, Piktel E, Gu X, Namiot Z, Kułakowska A, Savage PB, Bucki R 2015 Bactericidal activity and biocompatibility of ceragenin-coated magnetic nanoparticles. J Nanobiotechnology 13:1–11. doi:10.1186/s12951-014-0062-4. 25929281 PMC4458011

[14] Schindeler A, Nicole Y, Cheng TL, Sullivan K, Mikulec K, Peacock L, Matthews R, Little DG 2015 Local delivery of the cationic steroid antibiotic CSA-90 enables osseous union in a rat open fracture model of *Staphylococcus aureus* infection. J Bone Joint Surg Am 97:302–309. doi:10.2106/JBJS.N.00840. 25695982

[15] Williams DL, Haymond BS, Beck JP, Savage PB, Chaudhary V, Epperson RT, Kawaguchi B, Bloebaum RD 2012 *In vivo* efficacy of a silicone–cationic steroid antimicrobial coating to prevent implant-related infection. Biomaterials 33:8641–8656. doi:10.1016/j.biomaterials.2012.08.003. 22940221 PMC3588171

[16] Sinclair KD, Pham TX, Williams DL, Farnsworth RW, Loc-Carrillo CM, Bloebaum RD 2013 Model development for determining the efficacy of a combination coating for the prevention of perioperative device related infections: a pilot study. J Biomed Mater Res B Appl Biomater 101:1143–1153. doi:10.1002/jbm.b.32924. 23564717

[17] Raetz CR, Reynolds CM, Trent MS, Bishop RE 2007 Lipid A modification systems in Gram-negative bacteria. Annu Rev Biochem 76:295–329. doi:10.1146/annurev.biochem.76.010307.145803. 17362200 PMC2569861

[18] Gunn JS 2001 Bacterial modification of LPS and resistance to antimicrobial peptides. J Endotoxin Res 7:57–62. 11521084

[19] Pollard JE, Snarr J, Chaudhary V, Jennings JD, Shaw H, Christiansen B, Wright J, Jia W, Bishop RE, Savage PB 2012 *In vitro* evaluation of the potential for resistance development to ceragenin CSA-13. J Antimicrob Chemother 67:2665–2672. doi:10.1093/jac/dks276. 22899801 PMC3468081

[20] Richter SN, Frasson I, Bergo C, Parisi S, Cavallaro A, Palù G 2011 Transfer of KPC-2 carbapenemase from *Klebsiella pneumoniae* to *Escherichia coli* in a patient: first case in Europe. J Clin Microbiol 49:2040–2042. doi:10.1128/JCM.00133-11. 21411573 PMC3122676

[21] Goren MG, Carmeli Y, Schwaber MJ, Chmelnitsky I, Schechner V, Navon-Venezia S 2010 Transfer of carbapenem-resistant plasmid from *Klebsiella pneumoniae* ST258 to *Escherichia coli* in patient. Emerg Infect Dis 16:1014–1017. doi:10.3201/eid1606.091671. 20507761 PMC3086234

[22] Vila-Farres X, Callarisa AE, Gu X, Savage PB, Giralt E, Vila J 2015 CSA-131, a ceragenin active against colistin-resistant *Acinetobacter baumannii* and *Pseudomonas aeruginosa* clinical isolates. Int J Antimicrob Agents 46:568–571. doi:10.1016/j.ijantimicag.2015.08.003. 26395218

[23] Clinical and Laboratory Standards Institute. 2006 Methods for dilution antimicrobial susceptibility tests for bacteria that grow aerobically: approved standard, 7th ed CLSI document M7-A7 Clinical and Laboratory Standards Institute, Wayne, PA.

[24] Olajuyigbe OO, Afolayan AJ 2012 *In vitro* antibacterial and time-kill evaluation of the Erythrina caffra Thunb. extract against bacteria associated with diarrhoea. ScientificWorldJournal 2012:738314. doi:10.1100/2012/738314. 23213297 PMC3504411

[25] Hood MI, Becker KW, Roux CM, Dunman PM, Skaar EP 2013 Genetic determinants of intrinsic colistin tolerance in *Acinetobacter baumannii*. Infect Immun 81:542–551. doi:10.1128/IAI.00704-12. 23230287 PMC3553813

[26] Moskowitz SM, Ernst RK, Miller SI 2004 PmrAB, a two-component regulatory system of *Pseudomonas aeruginosa* that modulates resistance to cationic antimicrobial peptides and addition of aminoarabinose to lipid A. J Bacteriol 186:575–579. doi:10.1128/JB.186.2.575-579.2004. 14702327 PMC305751

[27] Llobet E, Martinez-Moliner V, Moranta D, Dahlstrom KM, Regueiro V, Tomas A, Cano V, Perez-Gutierrez C, Frank CG, Fernandez-Carrasco H, Insua JL, Salminen TA, Garmendia J, Bengoechea JA 2015 Deciphering tissue-induced *Klebsiella pneumoniae* lipid A structure. Proc Natl Acad Sci U S A 112:E6369–E6378. doi:10.1073/pnas.1508820112. 26578797 PMC4655541

[28] Gao R, Hu Y, Li Z, Sun J, Wang Q, Lin J, Ye H, Liu F, Srinivas S, Li D 2016 Dissemination and mechanism for the mcr-1 colistin resistance. PLoS Pathog 12:e1005957. doi:10.1371/journal.ppat.1005957. 27893854 PMC5125707

[29] Yeaman MR, Yount NY 2003 Mechanisms of antimicrobial peptide action and resistance. Pharmacol Rev 55:27–55. doi:10.1124/pr.55.1.2. 12615953

[30] Matamouros S, Miller SI 2015 *S. typhimurium* strategies to resist killing by cationic antimicrobial peptides. Biochim Biophys Acta 1848:3021–3025. doi:10.1016/j.bbamem.2015.01.013. 25644871 PMC4520786

[31] Yi EC, Hackett M 2000 Rapid isolation method for lipopolysaccharide and lipid A from Gram-negative bacteria. Analyst 125:651–656. doi:10.1039/b000368i. 10892021

[32] Arroyo LA, Herrera CM, Fernandez L, Hankins JV, Trent MS, Hancock RE 2011 The pmrCAB operon mediates polymyxin resistance in *Acinetobacter baumannii* ATCC 17978 and clinical isolates through phosphoethanolamine modification of lipid A. Antimicrob Agents Chemother 55:3743–3751. doi:10.1128/AAC.00256-11. 21646482 PMC3147623

[33] Clements A, Tull D, Jenney AW, Farn JL, Kim S-H, Bishop RE, McPhee JB, Hancock RE, Hartland EL, Pearse MJ 2007 Secondary acylation of *Klebsiella pneumoniae* lipopolysaccharide contributes to sensitivity to antibacterial peptides. J Biol Chem 282:15569–15577. doi:10.1074/jbc.M701454200. 17371870 PMC5007121

[34] Moffatt JH, Harper M, Harrison P, Hale JDF, Vinogradov E, Seemann T, Henry R, Crane B, St Michael F, Cox AD, Adler B, Nation RL, Li J, Boyce JD 2010 Colistin resistance in *Acinetobacter baumannii* is mediated by complete loss of lipopolysaccharide production. Antimicrob Agents Chemother 54:4971–4977. doi:10.1128/AAC.00834-10. 20855724 PMC2981238

[35] Girardello R, Visconde M, Cayô R, de Figueiredo RCBQ, da Silva Mori MA, Lincopan N, Gales AC 2017 Diversity of polymyxin resistance mechanisms among *Acinetobacter baumannii* clinical isolates. Diagn Microbiol Infect Dis 87:37–44. doi:10.1016/j.diagmicrobio.2016.10.011. 27776788

[36] Kao C, Lin X, Yi G, Zhang Y, Rowe-Magnus DA, Bush K 2016 Cathelicidin antimicrobial peptides with reduced activation of Toll-like receptor signaling have potent bactericidal activity against colistin-resistant bacteria. mBio 7:e01418-16. doi:10.1128/mBio.01418-16. 27651360 PMC5030359

[37] Conlon JM, Sonnevend A, Pál T, Vila-Farrés X 2012 Efficacy of six frog skin-derived antimicrobial peptides against colistin-resistant strains of the *Acinetobacter baumannii* group. Int J Antimicrob Agents 39:317–320. doi:10.1016/j.ijantimicag.2011.12.005. 22326566

