# Supplementary material for: Susceptibility of Colistin-Resistant, Gram-Negative Bacteria to Antimicrobial Peptides and Ceragenins
Source: Antimicrob Agents Chemother. 2017 Jul 25;61(8):e00292-17. doi: 10.1128/AAC.00292-17 (PMC5527650; doi:10.1128/AAC.00292-17)
Supplement: Supplemental material [file supp_61_8_e00292-17__index.html]

Supplemental material 

# Susceptibility of Colistin-Resistant, Gram-Negative Bacteria to Antimicrobial Peptides and Ceragenins

## Supplemental material

- Supplemental file 1 -

  Supplemental material

  PDF, 814K
