# Supplementary material for: Susceptibility of Colistin-Resistant, Gram-Negative Bacteria to Antimicrobial Peptides and Ceragenins
Source: Antimicrob Agents Chemother. 2017 Jul 25;61(8):e00292-17. doi: 10.1128/AAC.00292-17 (PMC5527650; doi:10.1128/AAC.00292-17)
Supplement: Supplemental material [file AAC.00292-17_zac008176423s1.pdf]

Supporting Information for Susceptibility of colistin-resistant, Gram-negative bacteria to antimicrobial peptides and ceragenins

Pages 2-3 Mass spectra (ESI, negative ion mode, Agilent 6230 Series TOF Spectrometer) of lipid A isolated from three clinical isolates of colistin-resistant *K. pneumoniae* (ARLG 1349, ARLG 1360 and ARLG 1389), from a colistin-susceptible strain (ATCC 13883) and from strains that were serially exposed to either colistin (Col-R) or CSA-131 (CSA-R).

Pages 4-6 Proposed structures for lipid A isolated from the indicated strains that match the mass spectral data. Changes from the parent structures are in red. Location of acylation (addition of a fatty acid) and phosphate ester formation may not reflect exact structure (i.e., mass spectral data do not provide positional information for modifications).

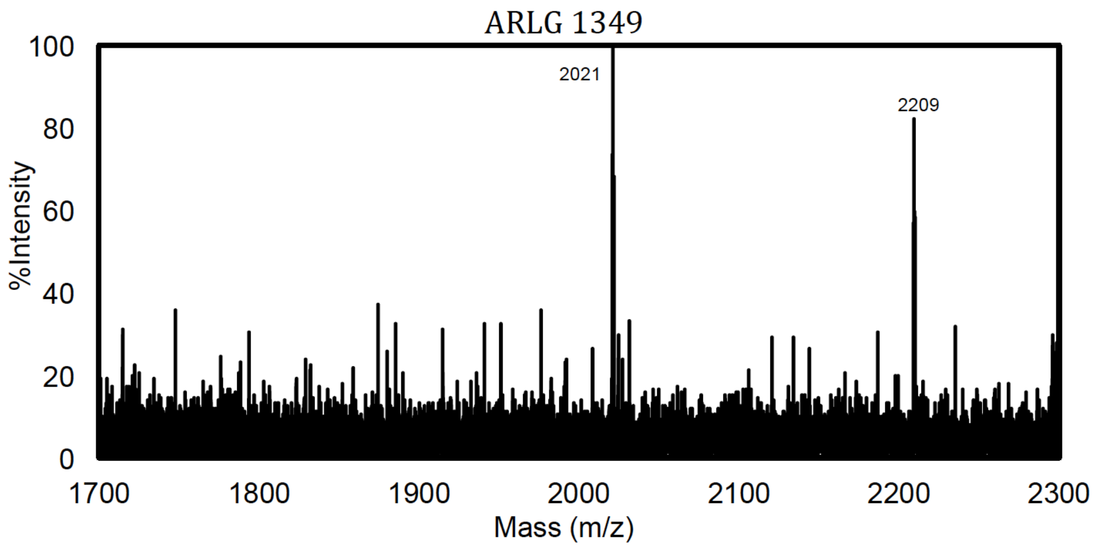
ARLG 1349
2021
2209
%Intensity
0
20
40
60
80
100
1700
1800
1900
2000
2100
2200
2300
Mass (m/z)

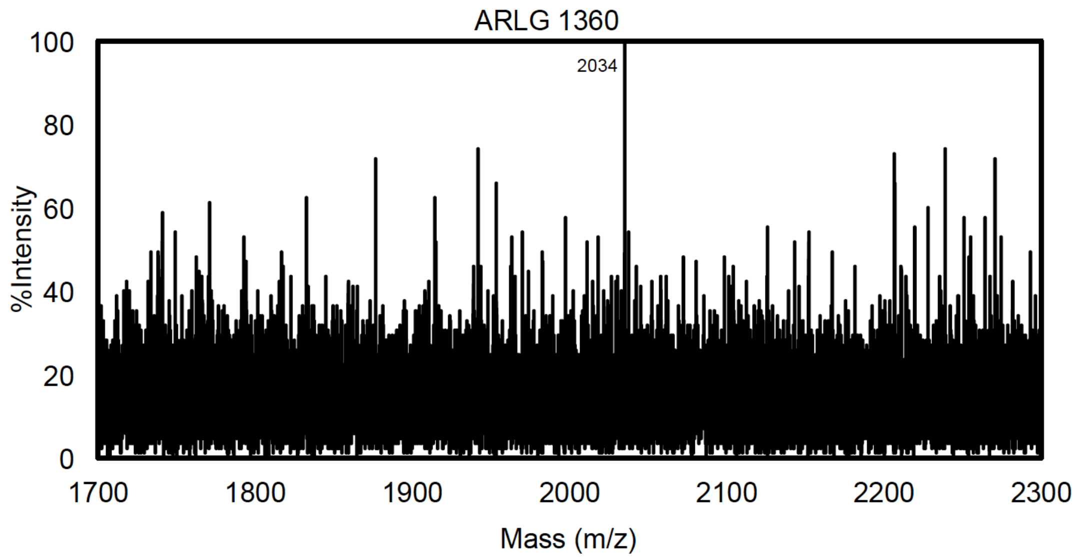
ARLG 1360
2034
%Intensity
0
20
40
60
80
100
1700
1800
1900
2000
2100
2200
2300
Mass (m/z)

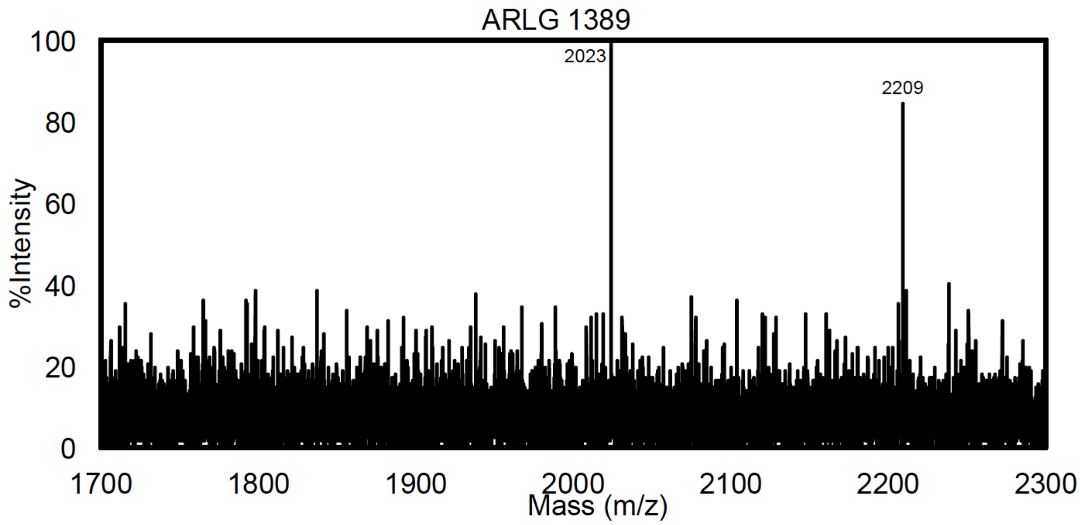
ARLG 1389
2023
2209
%Intensity
0
20
40
60
80
100
1700
1800
1900
2000
2100
2200
2300
Mass (m/z)

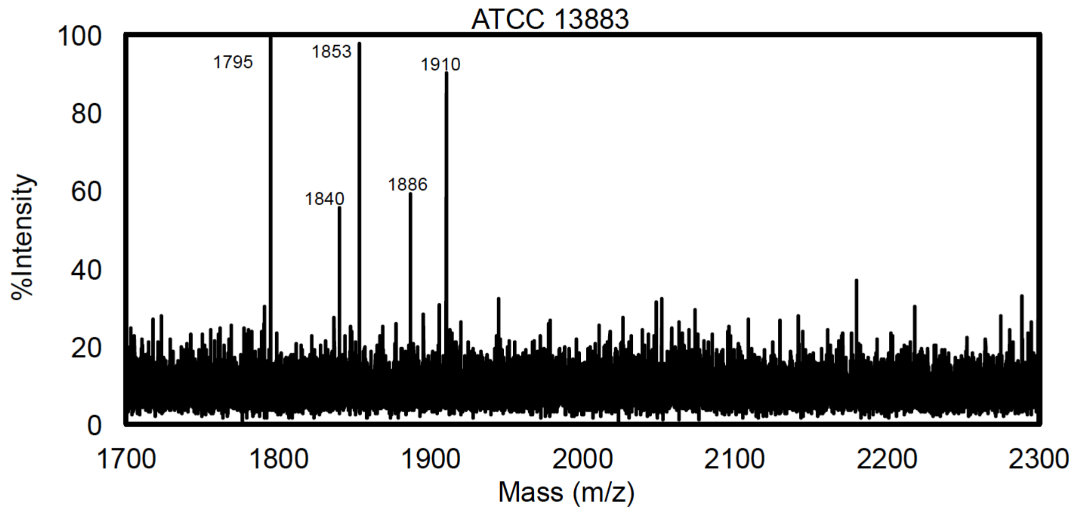
ATCC 13883
%Intensity
Mass (m/z)
1795
1853
1910
1840
1886
0
20
40
60
80
100
1700
1800
1900
2000
2100
2200
2300

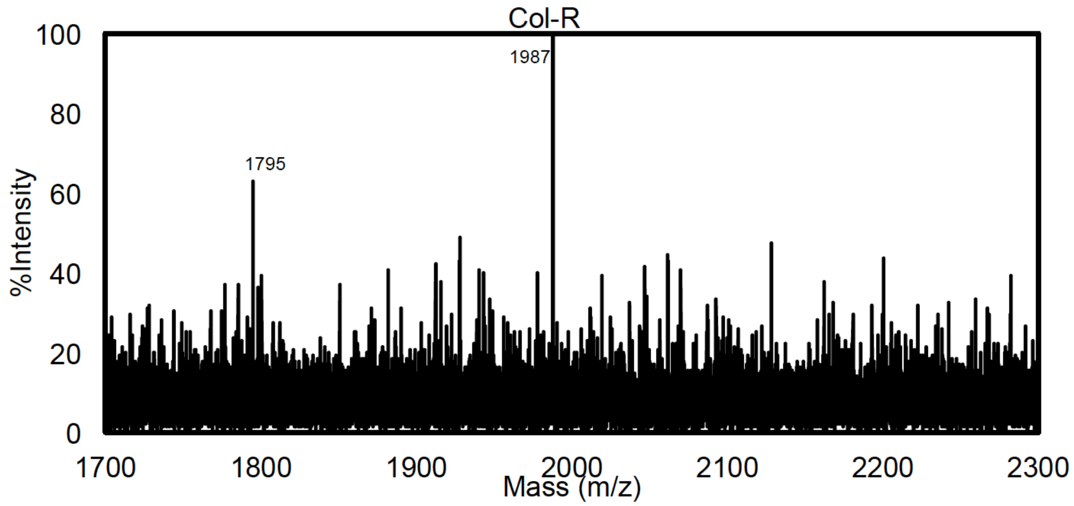
Col-R
%Intensity
Mass (m/z)
1987
1795
0
20
40
60
80
100
1700
1800
1900
2000
2100
2200
2300

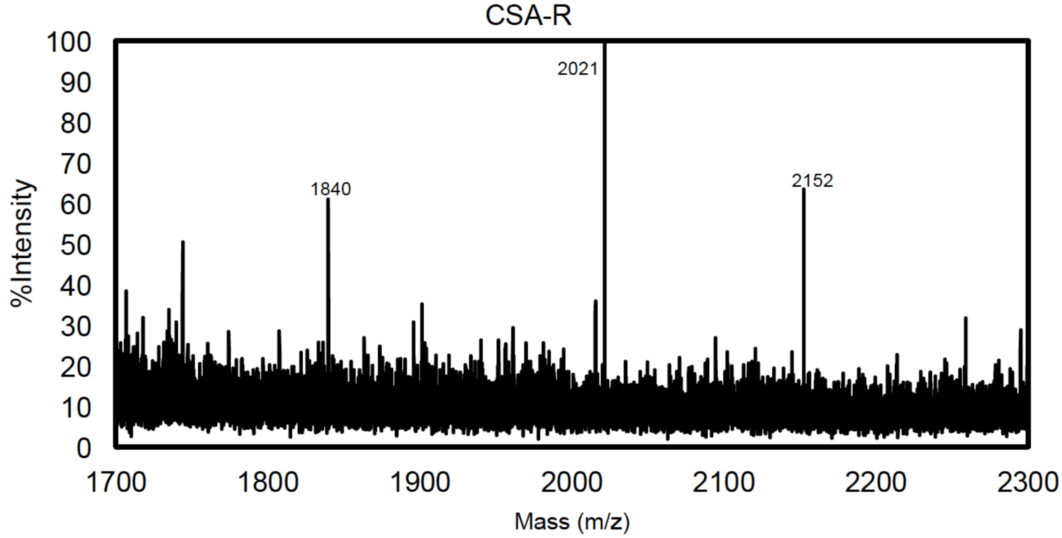
CSA-R
%Intensity
Mass (m/z)
2021
1840
2152
0
10
20
30
40
50
60
70
80
90
100
1700
1800
1900
2000
2100
2200
2300

ATCC 13883

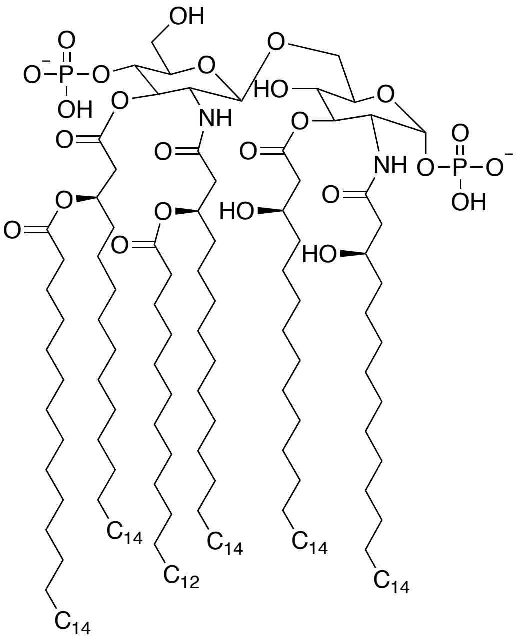

Exact Mass: 1795

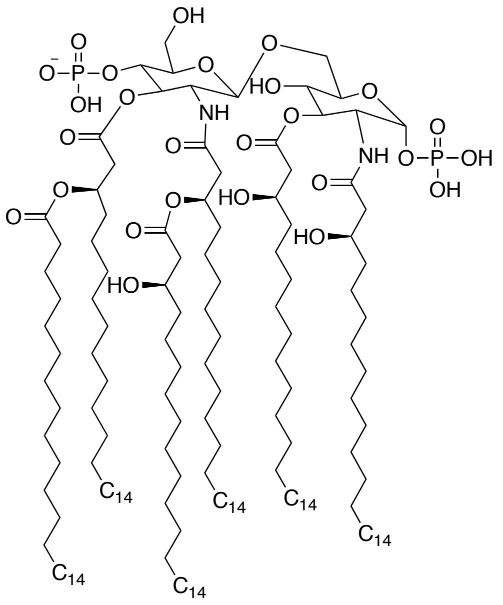

Exact Mass: 1840

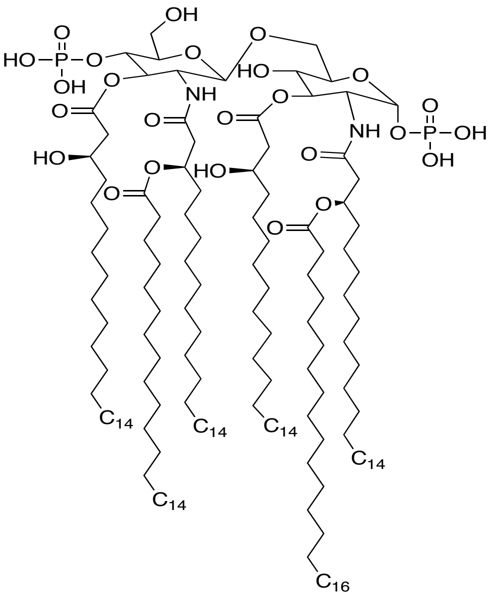

Exact Mass: 1853

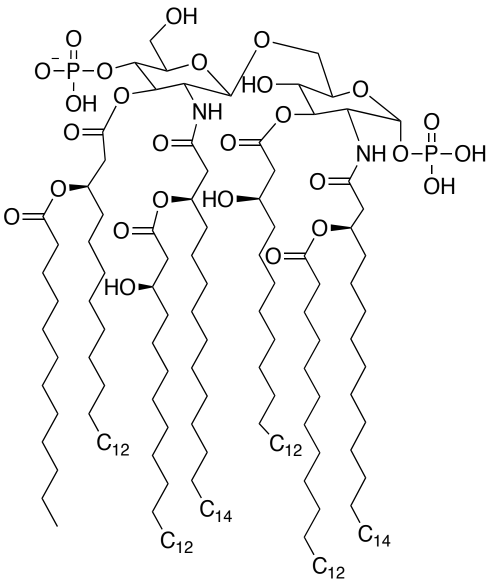

Exact Mass: 1910

ARLG 1349, ARLG 1389

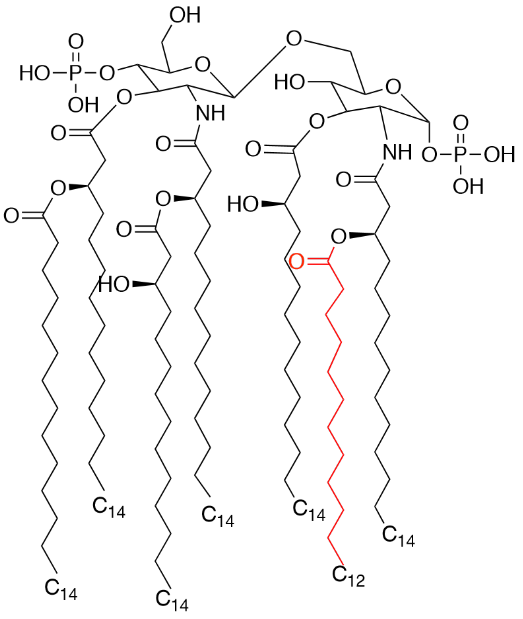

Exact Mass: 2023

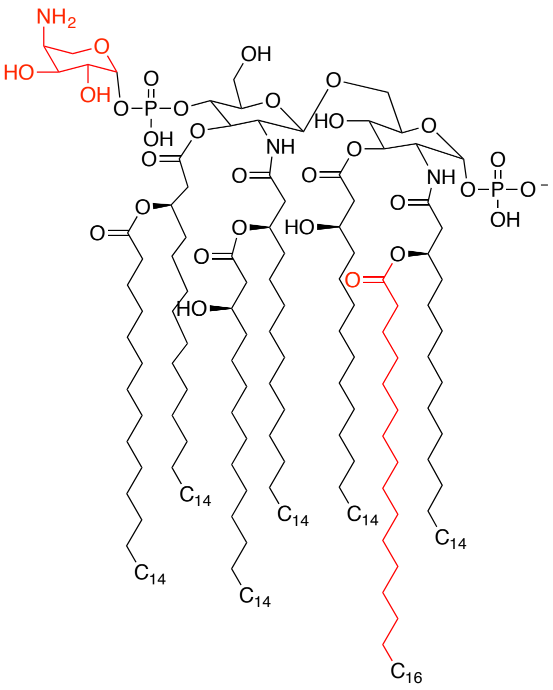

Exact Mass: 2209

ARLG 1360

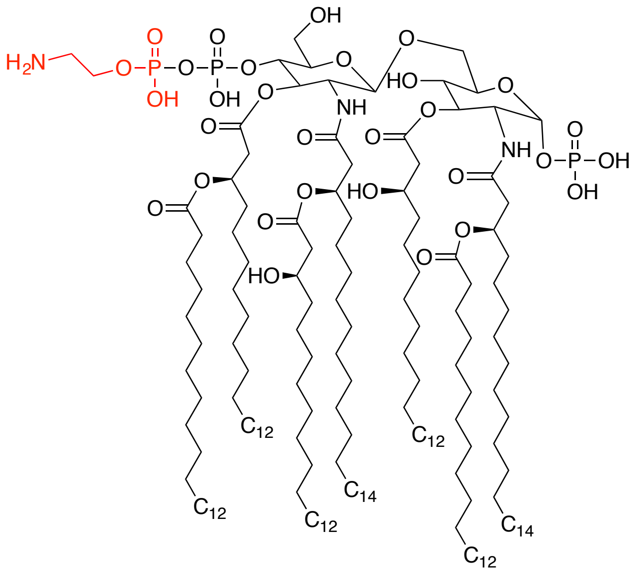

Exact Mass: 2034

Col-R (Colistin Resistant made by serial passaging)

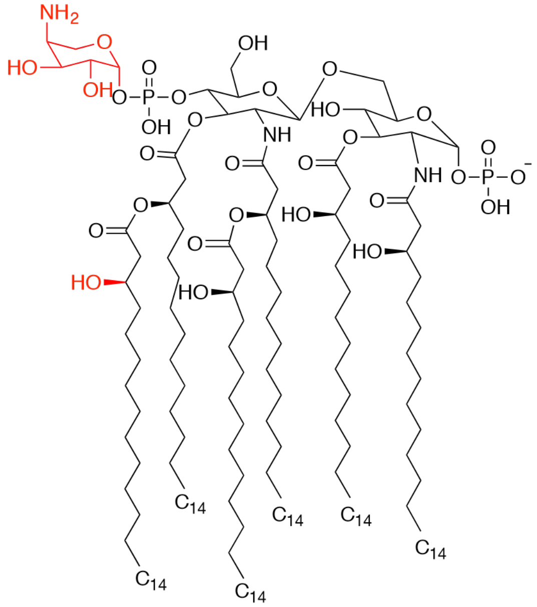

Exact Mass: 1987

CSA-R (CSA resistant made by serial passaging)

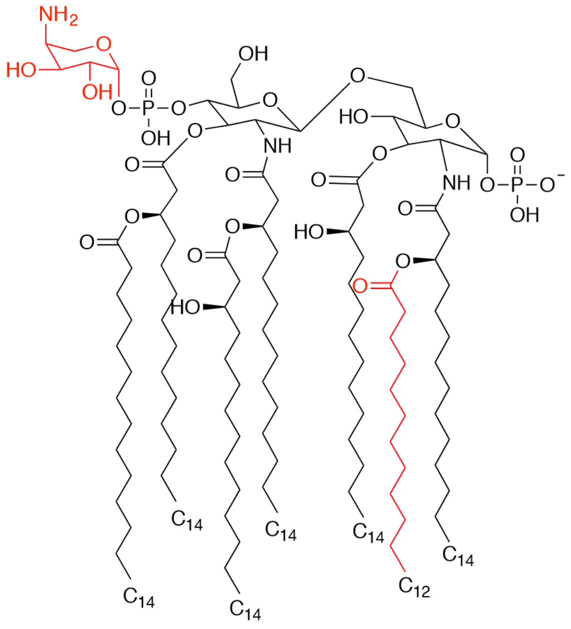

Exact Mass: 2153
